# Physical firmness increases structural alignment

**DOI:** 10.1038/s41598-023-27933-5

**Published:** 2023-01-17

**Authors:** Lorenzo Cecutti, Spike W. S. Lee

**Affiliations:** 1 Rotman School of Management, University of Toronto, Toronto, ON M5S 3E6 Canada; 2 Department of Psychology, University of Toronto, Toronto, ON M5S 3E6 Canada

**Keywords:** Human behaviour, Psychology

## Abstract

Raising one's jammed fist is not just a common pictorial representation of struggle against the establishment but turns out to reflect a deeper connection between sensorimotor states and beliefs. The present research investigated how physical firmness, manipulated through muscle tightening, might shape beliefs. The central hypothesis, structural alignment, was tested against two other competing predictions: content extremity and content matching. Three studies provided evidence supporting structural alignment but not content matching or extremity. Specifically, the correlation between intelligence and personality lay beliefs (Studies 1–2), and the correlation between individualizing and binding moral foundations (Study 3) increased when participants jammed their fist (Study 1) or clenched their jaw (Studies 2–3). These effects emerged in the absence of mean-level differences (which would have reflected content matching or extremity). Moreover, they did not seem attributable to response bias or tiredness. An additional study suggested decent rates of compliance with experimental instructions that were comparable between conditions. Overall, sensorimotor experiences such as physical firmness can align higher-order cognitions such as beliefs in ways that are distinct from prior demonstrations of embodied cognition effects.

## Introduction

"If there is no struggle, there is no progress. Those who profess to favor freedom, and yet depreciate agitation, are men who want crops without plowing up the ground. They want rain without thunder and lightning. They want the ocean without the awful roar of its many waters. This struggle may be a moral one; or it may be a physical one; or it may be both moral and physical; but it must be a struggle." – Fredrick Douglass (West India Emancipation Speech; August 3, 1857)

From the early twentieth century worker strikes in the U.S. to the more recent Black Lives Matter (BLM) demonstrations, the raised fist has been a perennial symbol of standing up against oppression and fighting for one's beliefs. But is this symbolic representation merely a random sociocultural custom? Or does it reflect some tractable connection between the mind and the body? We suggest a link between muscle contraction and structural alignment that the raised fist symbol might conceptually represent.

Historically, significant conflicts are often centered around clashing beliefs, from religion^1^ to political ideology^2^. Recent struggles also show how quickly peaceful protests in support of racial equality in the U.S.^3^ and democracy in Hong Kong^4^ run the risk of escalating into violence. Being in a "fight" mode activates the release of epinephrine, which increases muscle contractability^5^. Muscle tension sends feedback signals to the mind about its readiness to take action^6^. Could this relationship between motor signals and the brain also affect other cognitions, such as beliefs?

We propose that because muscle tension can be a signal to the brain that the body is about to act, it may activate a mindset of action readiness. Nuanced thinking, antithetical to action propensity, may be temporarily impaired to favor a more gestalt approach to cognitive work, thus leading people to engage in less nuanced or sophisticated thinking, which is often required to differentiate beliefs belonging to the same class or structure. Thus, our principal hypothesis is that (i) muscle firmness increases the consistency between structurally related beliefs. Specifically, assuming beliefs A and B are structurally related, the correlation between these two beliefs will increase with muscle contraction. We term this effect structural alignment.

However, the propensity towards taking action does not imply a shift in the core structure of beliefs or the views of the person experiencing the sensation of muscle firmness. Thus, we speculate that the actual belief strength and the nature of the belief will not shift. To investigate these, we formulate two more hypotheses. Specifically, (ii) muscle firmness could lead to more extreme views. Indeed, people could believe in A or B more strongly when experiencing muscle firmness. We call this effect content extremity. Or (iii) beliefs can match the state of experiencing muscle firmness. For example, intelligence can be perceived as being malleable or fixed. Therefore, when firming their muscles, people might be more inclined to believe that intelligence is fixed as this matches their physiological state. We call this content matching. We test for these three possibilities in each of the three studies and find that the physical sensation of muscle firmness (muscle contraction) increases alignment but not content extremity or matching.

### From muscle to mind

Bodily influences on cognitions have been previously documented in research on embodied cognition^7,8^. As an overarching perspective, embodied cognition challenges the notion that the mind operates as a separate entity from the body^9–11^. Specifically, cognition not only commands how our body reacts (e.g., motor acts such as nodding or shaking our head) but is also affected by these motor experiences and bodily sensations (e.g., nodding leads to more agreement than shaking our head)^12^. Even higher-order cognitions (e.g., moral beliefs) can be affected by bodily experiences. For example, moral judgments are associated with physical disgust^13–15^ and cleanliness^16^. Similarly, we argue that there is a connection between higher-order cognitions such as beliefs and lower-level physical sensations of muscle contraction.

While the existing literature has documented effects between bodily sensations and higher order cognitions, it has not explored any cognitive alignment effects. For example, a series of experiments connected the tactile experience of weight, hardness, and roughness (all different dimensions of touch) to social judgments about importance, rigidity in negotiations, and harshness in social interactions, respectively^17^. Another study focused on oral sensation and found that blocking subvocalization of brand names by giving participants something to chew on during advertisements reduced their likelihood of choosing advertised brands and attitudes towards them a week later^18^. Most relevant to our present focus, muscle contraction has been shown to increase participants' willpower and self-control^19^. In one of the previous studies, participants who firmed (vs. relaxed) their muscles drank more of a healthy but awful tasting medicine, which served as a behavioral indicator of stronger willpower and better self-control.

Unlike typical findings in this literature, content matching between the physical stimulus and the psychological effect (manifested through mean differences) is not our main prediction. Indeed, muscle firmness may be acting on how we process beliefs rather than on the specific content of these cognitions. Thus, the physical sensation of firmness may operate on a metacognitive level rather than directly on content level thoughts. If this is the case, we predict that the physical sensation of muscle contraction reinforces a belief system such that the correlation between two related beliefs will increase, thus increasing the coherence of separate beliefs belonging to the same belief system. This prediction, if supported, will be important for two reasons. (i) It will demonstrate a qualitatively different effect from classic embodiment demonstrations typically focusing on how motor sensory states influence thought content. (ii) It provides preliminary evidence that physical states, such as experiencing firmness, can have a metacognitive influence on cognitions such as beliefs.

We chose two classes of commonly held beliefs to test these effects: (1) lay beliefs about intelligence and personality^20,21^ and (2) moral beliefs^22,23^.

### Lay beliefs

Lay beliefs, or lay theories, are beliefs that people develop through individual experiences and upbringing about important factors in their lives, spanning a wide range of topics. In this research, we focus on intelligence^21^ and personality lay beliefs^20^. Beliefs about intelligence and personality differ in content but vary along one common continuum from fixed to malleable. Specifically, some people believe that intelligence or personality is fixed and cannot be changed (entity belief). In contrast, other people believe that intelligence or personality is malleable and can grow over time (incremental belief).

Intelligence and personality lay beliefs are essential as they have consequences for scholastic achievement^24,25^, how we judge others^20^ and relate in group settings^26^. Additionally, because these two lay beliefs share the common underlying idea of measuring how fixed (vs. malleable) a trait is, we can simultaneously test all three of our hypotheses against each other. If experiencing muscle firmness leads to structural alignment, then related beliefs are more likely to be coherent with each other. In operational terms:

#### H1a

The correlation between lay beliefs of intelligence and personality will be higher in the muscle firming than in the muscle relaxation (control) condition.

But if firming one's muscles makes one's beliefs more extreme, then participants' lay beliefs should approach the extremities of the scale. In operational terms:

#### H1b

The absolute difference between participants' lay belief scores and the scale midpoint will be higher in the muscle firming condition than in the muscle relaxation (control) condition.

Finally, if firming one's muscles matches one's beliefs to become more "firm," participants' intelligence and personality lay beliefs should become more fixed or entitative. In operational terms:

#### H1c

The mean entity score on the intelligence and personality lay beliefs scale will be higher in the muscle firming than in the muscle relaxation (control) condition.

### Moral beliefs

Early research on moral beliefs treated morality as a subset of beliefs about justice^27,28^. This view limited moral concerns to areas where an individual was treated unfairly or harmed by others. An example of unfairness includes receiving recognition and accolades for reasons other than merit, while an example of harm includes seeing someone suffering and not helping them. More recent developments in morality research highlighted three additional clusters of moral beliefs: loyalty, authority, and sanctity^29^. Loyalty is about the fulfillment of duties towards one's nation or ingroup. Authority concerns violations of laws and rules put in place by authority figures or disrespect towards those figures. Finally, sanctity is about preventing oneself from giving into carnal desires and cultivating and developing one's body and psyche.

These distinctions were formalized into an influential framework known as the moral foundations theory^22,23^. MFT classifies harm and fairness as individualizing foundations, while authority, loyalty, and purity as binding foundations^23^. This distinction is important because differences arise in the endorsement of these two clusters of foundations. For example, when looking at political differences, liberals mainly endorse individualizing foundations but not binding foundations as much, while conservatives moderately endorse both individualizing and binding foundations^30^. Overall, endorsement of individualizing foundations is only moderately correlated with the endorsement of binding foundations. Such moderate correlation leaves the capacity for the sensation of physical firmness to reinforce the overarching sense of morality and increase the alignment of moral beliefs. It also allows us to test our three competing hypotheses against each other.

Specifically, our central hypothesis is that if experiencing muscle firmness leads to structural alignment, then moral beliefs will remain the same. Thus we would expect the correlation between individualizing and biding moral foundations to be higher in the muscle firming condition.

#### H2a

The correlations between MFQ individualizing and biding foundations scores will be higher for participants in the muscle firming than in the muscle relaxation (control) condition.

Alternatively, if firming one's muscles leads one's moral beliefs towards extremity, then participants' judgments about whether something is relevant to the moral domain and whether it exemplifies something immoral should reach the extremity of the scales as a result of experiencing muscle firmness. If this were the case, we would expect the absolute value of the difference between the participant's MFQ scores and the scale midpoint to be higher in the muscle firming condition.

#### H2b

The absolute value of the difference between participants' MFQ scores and the scale midpoint will be higher for participants in the muscle firming than in the muscle relaxation (control) condition.

Finally, if experiencing physical firmness leads to a matching of people's moral beliefs, then we should expect an increase in the perception that both individualizing and binding foundations strongly pertain to the moral domain (judgment) and are morally relevant (relevance) when participants complete the moral foundations questionnaire (MFQ), thus resulting in a higher mean score on moral beliefs in the muscle firming condition.

#### H2c

The mean MFQ score will be higher for participants in the muscle firming than in the muscle relaxation (control) condition.

To test these competing hypotheses, we conducted three preregistered experiments. In studies 1 (https://osf.io/4km78/?view_only=bff926f7bf1b4fb09e5200b48e4e5322) and 2 (https://osf.io/7k9pq/?view_only=e6b4009107f844bf923caa99bd03974c), we study the effects of physical firmness on lay beliefs with different manipulations. Study 3 (https://osf.io/s3qfp/?view_only=f794fa139d0a4b22921b638b7d4d0c4c) extended the structural alignment effect to moral beliefs.

### Ethics declaration

The reported experiments were all conducted with approval from the Institutional Research Ethics Board at The University of Toronto. All methods were performed in accordance with the relevant guidelines and regulations. All participants provided informed consent to take part in the experiments. All participants were debriefed and explained the purpose of the studies and agreed to make their responses available for analysis.

## Study 1

This study aimed to demonstrate the structural alignment effect arising from the experience of physical firmness. We tested our central hypothesis, structural alignment H1a, against content extremity H1b and content matching H1c. This study’s preregistration of methods and analyses can be found at https://osf.io/4km78/?view_only=bff926f7bf1b4fb09e5200b48e4e5322.

### Methods

#### Participants

Three hundred twelve participants were recruited through Amazon Mechanical Turk and provided a small monetary compensation for participating in this study. The number of participants recruited was determined through a priori power analysis based on similar effects we found in exploratory studies. Through G*Power, we calculated that to obtain a similar effect in a 2-condition between-subjects design with 0.8 power and 0.05 alpha, we needed a total sample of 260 participants. Due to the attention check filters and possible dropouts, we collected 20% more for a total of 312 participants. In the end, out of 312 participants, 248 passed the attention check.

#### Procedure

As part of the cover story for this study, participants were told to measure their pulse by placing their dominant hand's index and middle fingers onto the wrist of their nondominant hand.

Firmness was manipulated by asking participants to either squeeze their nondominant hand into a fist (squeeze condition) or keep an open palm (hold condition) while performing the pulse measurement. The pulse measurements happened twice in the study. The first measurement occurred immediately after the manipulation instructions and the second towards the end of the survey before the demographics section. Importantly, in between measures, participants were instructed to keep their nondominant hand in position to ensure that the manipulation continued while participants completed the main task and dependent variable. Importantly, this allowed participants to simultaneously use their free dominant hand to click on responses and advance through the survey. This manipulation setup resulted in a 2 (firmness: squeeze vs. hold) between-subjects design.

After the briefing and manipulation sections, participants read a short article ostensibly reporting on scientific findings describing intelligence as being fixed and then rated their agreement with the article. Then they completed a scale about personality lay beliefs^20^. The correlation between article agreement and personality lay belief was the dependent variable for this study. Agreement with the article was worded as follows. *"According to your own opinion, a person's intelligence is…"* "*1* = *Very much malleable and can change; 7* = *Very much fixed and cannot change*". A low score indicates low agreement, whereas a high score indicates high agreement. The personality lay beliefs^20^ scale is made up of 6 items such as *"the kind of person someone is, is something very basic about them and it can't be changed very much"* and *"people can do things differently, but the important parts of who they are can't really be changed"* ranging from "*1* = *strongly disagree*; *6* = *strongly agree*".

After that, participants were asked to take the second pulse measurement, given a manipulation check, and asked about their level of tiredness and mood. The manipulation check was worded "*To what extent did the heartbeat task involving your hand give you a sense of firmness?"* where *"0* = *No sense of firmness at all; 6* = *Very strong sense of firmness"*. Finally, they completed an attention check and demographic information and were debriefed.

### Results

#### Manipulation check

We ran an ANOVA with our manipulation as the independent variable and the manipulation check measure as the dependent variable. The manipulation check revealed that participants in the treatment condition experienced significantly more firmness (*M*_*squeeze*_ = 5.00, *SD*_*squeeze*_ = 1.48) than participants in the control condition (*M*_*hold*_ = 3.95, *SD*_*hold*_ = 1.71; *F*(1,246) = 26.76, *p* < 0.001). Thus, our manipulation successfully elicited a feeling of physical firmness in participants.

#### Hypothesis testing

##### H1a: structural alignment analysis

To test for structural alignment, we first computed the correlation between article agreement and personality lay beliefs for each condition. Then, we compared these two scores by running a correlation difference test. Supporting H1a, we found that the correlation between article attitude and personality lay beliefs was significantly higher in the squeeze condition (*r*(128) = 0.66) than the hold condition (*r*(120) = 0.49; *z* = 2.04, *p* = 0.04). The results provided evidence for structural alignment when participants squeezed their fists.

##### H1b: content extremity analysis

To test H1b, we computed the absolute value of the difference of each participant's score on the measure indicating agreement with the article and the personality lay belief scale and subtracted it from the scale midpoint (4 and 3.5 respectively). Then we ran two ANOVAs comparing these scores between conditions. Participants’ own opinions about intelligence lay beliefs (*F*(1,246) = 0.19, *p* = 0.66) or responses on the personality lay beliefs scale (*F*(1,246) = 0.97, *p* = 0.33) did not become more extreme. These results show that participants' answers on the personality lay beliefs scale did not become more extreme due to squeezing their fist.

##### H1c: content matching analysis

Finally, to test for content matching, we ran two ANOVAs with article agreement and personality lay beliefs mean scores as dependent variables and the firmness manipulation as the independent variable. We found that conditions did not differ in terms of participant’s own beliefs about intelligence after reading the article (*M*_*squeeze*_ = 3.87, *SD*_*squeeze*_ = 1.84; *M*_*hold*_ = 3.75, *SD*_*hold*_ = 1.76; *F*(1,246) = 0.26, *p* = 0.61) and their answer to the personality lay beliefs scale (*M*_*squeeze*_ = 3.27, *SD*_*squeeze*_ = 1.19; *M*_*hold*_ = 3.18, *SD*_*hold*_ = 1.11; *F*(1,246) = 0.32, *p* = 0.57). This shows that there is no evidence to support the hypothesis that firmness can alter or sway participants’ beliefs.

#### Controls

##### Tiredness

The structural alignment effect was found regardless of participants' level of tiredness. Specifically, we categorized participants into the high-tiredness group (i.e., tiredness equal or above the scale mean score) and low-tiredness group (i.e., tiredness below the scale mean score) and ran a correlation difference on our dependent variables. We found that the correlation between article attitude and personality lay beliefs did not differ significantly between the low-tiredness (*r*(153) = 0.59) and high-tiredness groups (*r*(95) = 0.58), *z* = 0.07, *p* = 0.47, suggesting that tiredness did not drive our results.

##### Mood

Additional analysis did not reveal that our manipulation was driving any differences in positive (*M*_*squeeze*_ = 5.20, *SD*_*squeeze*_ = 1.26; *M*_*hold*_ = 5.23, *SD*_*hold*_ = 1.34; *F*(1,246) = 0.02, *p* = 0.90) or negative mood (*M*_*squeeze*_ = 3.17, *SD*_*squeeze*_ = 1.45; *M*_*hold*_ = 2.94, *SD*_*hold*_ = 1.35; *F*(1,246) = 1.43, *p* = 0.23). Thus, the manipulation did not alter participants’ mood during the study, which could have influenced our results.

##### Response mode

Another potential issue is that participants may be distracted by the fist squeezing manipulation. If this is the case, they may answer in a way that seems consistent across article agreement, and personality lay beliefs, but that does not indicate structural alignment of beliefs. To alleviate this response mode concern, we conducted Levene's test for equality of variance. This test compares variance in responses across two conditions. If the variance is significantly lower in the manipulation condition, response mode due to inattention may be a concern. We found that for both article agreement (*F*(1,246) = 0.17, *p* = 0.68) and personality (*F*(1, 246) = 0.96, *p* = 0.33) variance scores did not significantly differ. Thus, we concluded that response mode may not have influenced the results in this study.

### Discussion

The results for Study 1 showed support for H1a. Specifically, participants' scores for article agreement and personality lay beliefs were correlated more strongly when they experienced physical firmness. These results suggest evidence in favor of the alignment hypothesis (H1a). In contrast, this study did not find evidence supporting the content extremity hypothesis (H1b) or the content matching hypothesis (H1c).

Study 1 manipulated firmness using muscle contraction experienced through squeezing one's fist. This could affect beliefs due to the associations the raised fist symbol has in our society. Our cover story did not mention those associations. Thus, it is less likely that participants came to those associations consciously. However, to alleviate such concern and extend the effect to different kinds of muscle contraction, we manipulated physical firmness through jaw clenching by contracting mandibular muscles in the following study.

## Study 2

This study's preregistration can be found at https://osf.io/7k9pq/?view_only=e6b4009107f844bf923caa99bd03974c. The methods and procedures for this study are identical to those in Study 1. The only difference is how physical firmness was manipulated. Specifically, Study 2’s manipulation of physical firmness involved asking participants to clench their jaw by contracting mandibular muscles (instead of squeezing their fist in Study 1).

### Methods

#### Participants

Three hundred fifteen participants were recruited through Amazon Mechanical Turk and provided a small monetary compensation for participating in this study. The number of participants recruited was determined through the same a priori power analysis described in Study 1. Out of 315 participants, 241 passed the attention check.

#### Procedure

In this study, firmness is elicited by asking participants to clench their jaw, while in the control condition, they were told to place their tongue against their upper teeth, which was meant to relax their jaw muscles. This design results in a 2 (firmness: clench vs. control) between subject design. Participants were ostensibly told that this is an informal medical test to measure TMJ (temporomandibular joint) dysfunction. Because of this, as part of the cover story, we asked them to report their pain level after completing the dependent variable instead of measuring their pulse. Additionally, we modified the wording of our manipulation check to reflect the cover story changes *"To what extent did the Temporomandibular Test involving your jaw give you a sense of firmness?"* where *"0* = *No sense of firmness at all; 6* = *Very strong sense of firmness."*

### Results

#### Manipulation check

We ran an ANOVA with our manipulation as the independent variable and the manipulation check measure as the dependent variable. The manipulation check revealed that participants in the treatment condition experienced significantly more firmness (*M*_*squeeze*_ = 4.90, *SD*_*squeeze*_ = 1.63) than participants in the control condition (*M*_*hold*_ = 3.56, *SD*_*hold*_ = 1.53; *F*(1,239) = 43.60, *p* < 0.001). Thus, our manipulation successfully elicited a feeling of physical firmness in participants.

#### Hypothesis testing

##### H1a: structural alignment analysis

The results from this study provide further support for H1a. Specifically, the correlation between article attitude and personality lay beliefs was significantly higher in the squeeze condition (*r*(117) = 0.75) than the hold condition (*r*(124) = 0.59; *z* = 2.25, *p* = 0.02).

##### H1b: content extremity analysis

Replicating findings from Study 1, in Study 2 we also did not find evidence of content extremity (article agreement: *F*(1,239) = 0.42, *p* = 0.52; personality lab beliefs: *F*(1,239) = 0.11, *p* = 0.74).

##### H1c: content matching analysis

Again confirming that firmness does not sway beliefs, conditions did not differ in terms of their mean answers to attitudes towards the article (*M*_*clench*_ = 3.85, *SD*_*clench*_ = 1.91; *M*_*control*_ = 3.73, *SD*_*control*_ = 1.79; *F*(1,239) = 0.25, *p* = 0.62) and their personality lay beliefs (*M*_*clench*_ = 3.21, *SD*_*clench*_ = 1.20; *M*_*control*_ = 3.23, *SD*_*control*_ = 1.15; *F*(1,239) = 0.01, *p* = 0.94).

#### Controls

##### Tiredness

Tiredness did not play a role in this study. The correlation between article attitude and personality lay beliefs was not significantly different in the low tired (*r*(127) = 0.66) than the high tired condition (*r*(114) = 0.69; *z* = − 0.39, *p* = 0.7). Suggesting that tiredness did not influence the main result in this study.

##### Mood

Additional analysis did not reveal that our manipulation was driving any differences in positive (*M*_*squeeze*_ = 5.10, *SD*_*squeeze*_ = 1.34; *M*_*hold*_ = 4.98, *SD*_*hold*_ = 1.39; *F*(1,239) = 0.52, *p* = 0.47) or negative mood (*M*_*squeeze*_ = 2.90, *SD*_*squeeze*_ = 1.67; *M*_*hold*_ = 2.82, *SD*_*hold*_ = 1.62; *F*(1, 239) = 0.13, *p* = 0.72). Thus, the manipulation did not alter participants’ mood during the study, which could have influenced our results.

##### Response mode

Finally, we conducted Levene's test for equality of variance to test for possible response mode concerns. We found that for both article agreement (*F*(1, 239) = 0.77, *p* = 0.38) and personality (*F*(1, 239) = 0.57, *p* = 0.45) variance scores did not significantly differ. Thus, response mode due to inattention is not a concern in this study.

### Discussion

Study 2 further supports structural alignment, suggesting that firmness does not seem to lead to belief matching or extremity. Additionally, it demonstrates that contraction of muscles not belonging to our forearm or hands can also lead to the effect of structural alignment. Indeed, muscle firmness was manipulated by asking participants to clench their jaw, which involved their facial muscles, thus freeing their hands. Lastly, Study 2 also shows that tiredness, mood, and response mode do not drive this effect. However, all studies so far have focused on lay beliefs about intelligence and personality. So, does the firmness effect extend to other classes of beliefs? Study 3 tested this possibility in the context of moral beliefs.

## Study 3

This study's preregistration can be found at https://osf.io/s3qfp/?view_only=f794fa139d0a4b22921b638b7d4d0c4c.

The goal of this study is to investigate whether physical firmness can influence moral beliefs. Specifically, we are interested in observing two classes of moral beliefs: binding and individualizing moral foundations. These are distinct clusters of beliefs, but they are structurally related to the more general class, moral beliefs. If physical firmness increases structural alignment, then we should observe a higher correlation between binding and individualizing moral foundations in the muscle firming condition than in the control condition.

### Methods

#### Participants

Six hundred seventy three participants were recruited through Amazon Mechanical Turk and provided a small monetary compensation for participating in this study. The number of participants recruited was determined through a priori power analysis.

We preregistered a two step data collection strategy to account for a possible smaller effect size than anticipated. In the first step of data collection, we predicted a similar effect size to the one found in a previous exploratory study. If the first step of data collection proved insufficient to detect an effect, we proceeded to step two, where we predicted an effect 75% the size of the one we found initially. To calculate the sample needed for step two, we used the difference in correlations from the previous study as an effect size estimate. We multiplied this estimate by 0.75 (q = 0.24). Through G*Power, we calculated that to obtain a similar effect in a 2-condition between-subjects design with 0.8 power and 0.05 alpha, we should target a total sample of 548 participants. However, due to the attention check filters and possible dropouts, we targeted to collect 20% more for a total of 658 participants.

A total of 673 participants answered our survey, and 570 passed the preregistered attention check and time checks (completing the survey between 100 s and 33 min, equaling the mean plus two times the standard deviation).

#### Procedure

First, physical firmness was manipulated through jaw clenching. This manipulation resulted in a 2 (firmness: clench vs. control) between subject design. Participants were then given the moral foundations questionnaire (MFQ) ^22^. This questionnaire included both statements about moral judgments (e.g., *"Compassion for those who are suffering is the most crucial virtue," "One of the worst things a person could do is hurt a defenseless animal"*) and moral relevance (e.g., *"Whether or not someone suffered emotionally," "Whether or not someone cared for someone weak or vulnerable"*). Participants answered these questions on a scale of "*1—strongly disagree; 6—strongly agree"* for moral judgments and *"1—not at all relevant; 6—extremely relevant"* for moral relevance items. They were then asked to rate their pain level as per the cover story and complete the manipulation check. This manipulation check was identical to the one described in Study 2. Finally, they completed the attention check measure and demographic information and were debriefed.

### Results

#### Manipulation check

We ran an ANOVA with our manipulation as the independent variable and the manipulation check measure as the dependent variable. The manipulation check revealed that participants in the treatment condition experienced significantly more firmness (*M*_*squeeze*_ = 4.85, *SD*_*squeeze*_ = 1.49) than participants in the control condition (*M*_*hold*_ = 3.49, *SD*_*hold*_ = 1.71; *F*(1, 568) = 102.71, *p* < 0.001). Thus, our manipulation successfully elicited a feeling of physical firmness in participants.

#### Hypothesis testing

##### H2a: structural alignment analysis

Analysis revealed evidence suggesting structural alignment, even for beliefs about morality (H2a). Specifically, we examined moral relevance and moral judgments subscales, which resulted in a 5 × 5 matrix of correlations for each condition (see Table 1).

**Table 1:** MFQ judgment and relevance correlations.

MFQ Judgment	MFQ relevance				
	Harm	Fairness	Ingroup	Authority	Purity
Jaw Clench *n* = 282					
Harm	0.44**	0.34**	0.26**	0.27**	0.19**
Fairness	0.35**	0.45**	0.23**	0.25**	**0.11**
Ingroup	**0.01**	**0.03**	0.51**	0.51**	0.56**
Authority	**0.02**	**0.07**	0.46**	0.48**	0.52**
Purity	**0.04**	**0.06**	0.44**	0.50**	0.71**
Hold *n* = 288					
Harm	0.41**	0.35**	0.14*	0.16**	0.18**
Fairness	0.32**	0.40**	**0.08**	0.12*	**0.10**
Ingroup	− 0.12*	− 0.13*	0.50**	0.49**	0.45**
Authority	**0.03**	**−0.05**	0.55**	0.59**	0.54**
Purity	**0.00**	**− 0.03**	0.48**	0.58**	0.79**

We could not run simple correlation difference tests because of the number of correlations we needed to compare. Also, averaging the correlations in each condition would have reduced the number of observations we could compare, thus inflating the probability of a type II error. Therefore, using the off-diagonal correlations in this table (which correspond to correlations between individualizing and binding foundations), we compared clench and control conditions looking for correlation differences (24 correlations). We ran a t-test using the standardized correlation scores as data inputs. We tested for differences between correlations belonging to clenching and holding conditions.

Bold Font indicates correlations that have been standardized (z-transformed) and compared between conditions.

*Indicates significance at the 0.05 level.

**Indicates significance at the 0.01 level.

The correlation between binding and individualizing foundations was overall higher when participants clenched their jaw (*M*_*clench*_ = 0.13, *SD*_*clench*_ = 0.01) compared to the control condition (*M*_*clench*_ = 0.04 *SD*_*clench*_ = 0.01; t(22) = 2.08, p = 0.049)*.* These results suggest that physical firmness increases moral structural alignment.

##### H2b: content extremity analysis

Additionally, when testing for possible extremity effects, we did not find evidence supporting H2c. Specifically, we ran two ANOVA tests for the absolute value of each participant's individualizing and binding score minus the scale midpoint (3.5). We did not find significant differences between conditions for both individualizing (*F*(1, 568) = 0.12, *p* = 0.73) and binding foundations (*F*(1, 568) = 0.22, *p* = 0.64).

##### H2c: content matching analysis

To test for mean differences between firmness conditions in moral beliefs we ran an ANOVA predicting individualizing and binding foundations. Regarding individualizing foundations we did not find any difference between clench (*M*_*clench*_ = 4.62, *SD*_*clench*_ = 0.71) and control conditions (*M*_*control*_ = 4.64, *SD*_*control*_ = 0.69; *F*(1,568) = 0.08, *p* = 0.78). Likewise, we did not find any differences between clench (*M*_*clench*_ = 3.78, *SD*_*clench*_ = 0.90) and control condition (*M*_*control*_ = 3.74, *SD*_*control*_ = 0.95; *F*(1, 568) = 0.22, *p* = 0.64) when predicting binding foundations.

### Discussion

These results show that, when participants experienced muscle firmness, the overall correlation between binding and individualizing foundations was higher, providing evidence for the structural alignment hypothesis. Could this effect be due to higher engagement? If participants were more engaged as a result of physical firmness, this should simply amplify their existing views, leading to content extremity, for which we found no support. Instead, physical firmness led to an increase in correlation between two sets of moral foundations that tend to be endorsed by people with opposing ideologies. Indeed, because structural alignment is the focus on the shared structure of two beliefs and both binding and individualizing foundations share the same core concern about moral good or evil, participants experiencing firmness rated these two distinct classes of moral beliefs more similarly as they were more focused on this core similarity. This pattern of results could not be explained by engagement. Nor could the conceptually similar results in Studies 1 and 2.

A limitation, present throughout the studies discussed so far is that all the studies were conducted online and, thus, we could not observe whether participants complied with our manipulation task or not. To address this concern, we ran an additional study encouraging participants’ honest self report.

## Additional study: manipulation compliance

This posttest’s preregistration can be found at https://osf.io/3eyfw/?view_only=74500354b162498699bb3da2816cf59b

The goal of this short study is to empirically address a potential general methodological concern. Specifically, this study investigates whether participants to our online studies follow the experimental manipulation instructions. Indeed, it is possible that i) participants have a generally low compliance rate and do not perform the task when asked to and that ii) participants may be selectively performing (or avoiding performing) the manipulation task depending on their condition assignment. This would diminish the validity of our conclusions. Thus, we decided to run this additional study on the same online population we ran our previous studies and check for compliance.

### Methods

#### Participants

Two hundred fifty participants were recruited through Amazon Mechanical Turk and provided a small monetary compensation for participating in this study. A total of 254 participants completed our survey.

#### Procedure

First, we administered the jaw clench manipulation of physical firmness and randomly assigned participants to one of two conditions: clench or control. Participants were subsequently told the purpose of the study and instructed that their compensation would not be affected by how they answered any of our questions. Subsequently, we asked them to indicate with full honesty whether they had completed the task following our instructions. Participants could either indicate that they performed the manipulation task (“yes”) or indicate that they did not (“no”). This was followed by a brief demographics section and debriefing.

### Results

Out of 254 participants, 3 participants in the clench condition indicated that they did not follow the instructions, and no participant in the control condition indicated they did not follow the instructions. We ran a Chi-square analysis to test whether there was any significant difference between conditions in instruction compliance rates. We found that there was no significant difference in compliance rates between conditions χ^2^(1, *N* = 254) = 0.25, *p* > 0.05.

### Discussion

The small (1%) rate of participants that indicated that they did not comply with our manipulation instructions, paired with the evidence that there was no significant difference between conditions in terms of compliance rates, suggested that the results in prior studies were unlikely to be driven by an experimental artifact involving task instruction compliance for online studies.

## General discussion

Three studies show that experiencing physical firmness through muscle contraction increases structural alignment in the form of a higher correlation between substantively distinct but structurally related beliefs. Studies 1 and 2 show that the structural alignment effect arises when participants jam their nondominant hand fist or clench their jaw, different manipulations of physical firmness. Additionally, it consistently demonstrates that physical firmness elicits structural alignment and not matching or extremity of beliefs. Finally, Study 3 extends the effect of physical firmness to moral beliefs, showing that binding and individualizing moral foundations correlate more strongly when participants clench their jaw.

These findings advance theoretical understanding on two fronts. (i) They demonstrate structural alignment and distinguish it from matching and extremity. (ii) More broadly, they highlight connections between the bodily sensation of firmness and higher cognitions such as beliefs. We elaborate on both points below.

Most commonly, beliefs shift from one view to another (match) or become more extreme (extremity). However, the effect we draw attention to in these studies is more subtle. Structural alignment happens when conceptually distinct but structurally related beliefs become more correlated. We can interpret this alignment as being akin to gaining confidence over one's beliefs without those beliefs necessarily getting more extreme or changing. The present work illustrates this effect and shows how physical action, precisely the sensation of muscle firmness, can lead to structural alignment in areas of lay beliefs and morality. These two areas are of particular importance as they determine a variety of judgments and behaviors in people's lives, as discussed earlier.

Besides theoretical implications, this research suggests three practical recommendations for understanding beliefs, sensory experiences, and experimental methodology. First, people interested in beliefs will note that this research shows that beliefs might be more malleable than commonly assumed. For example, the simple incidental sensation of muscle firmness, activated through jaw clenching, can result in structural alignment for worldviews and morality. In practice, this means that people should become aware of how incidental sensations could impact their beliefs and more broadly view their own beliefs as something less than perfectly stable. Second, regarding sensorimotor experiences, it is generally well established that how we feel can impact how rigid our muscles feel. For example, when feeling stressed, we may engage in jaw clenching and involuntarily firm our shoulders. However, it is a lesser known fact that these physical sensations may affect higher order cognitions such as beliefs. Armed with this knowledge we should pay attention to the gestures and bodily sensations we engage in while debating about our beliefs or risk to be influenced by them without intending to do so. This, in turn, highlights our third practical recommendation about experimental methodology. If incidental sensorimotor experiences, such as physical firmness, can impact beliefs, then it is also possible that they may impact other cognitions. Thus, it is important to account for these incidental effects when conducting research that may involve movement or muscle contraction. For example, it may be important to monitor participants’ bodily posture and muscle contraction when running experiments investigating beliefs.

While the studies do make nuanced theoretical distinctions and offer practical recommendations, they are not without limitations. First, our experimental paradigm offers high internal, but low external, validity for our findings. Second, there may be additional ways to elicit physical firmness and thus lead to structural alignment, which were not tested in our studies. Both limitations are discussed in turn below.

The experimental paradigm used in all three studies allowed us to cleanly manipulate physical firmness. Indeed, the only feature that changed between control and treatment conditions were whether isometric muscle contraction was elicited (treatment) or not (control). Every other aspect such as posture or hand placement were kept identical in both conditions. This high degree of control grants greater certainty that muscle firmness leads to structural alignment, thus increasing the internal validity of our studies. However, the degree of control offered by the manipulations makes them unlikely to naturally occur in daily life and thus lowers the degree of external validity of our studies. We believe that the value of cleanly pinpointing the role of physical firmness in eliciting structural alignment justifies the tradeoff, but this remains a limitation of our studies.

In this research we showed two distinct ways to elicit physical firmness, fist jamming and jaw clenching. Other ways of eliciting physical firmness may also give rise to the structural alignment effect. Early pretests in our lab using a hand strengthener device showed similar results of increasing the correlation between intelligence and personality lay beliefs. Additionally, we considered using calf muscle contraction as another way to elicit physical firmness. Eventually, because of the ease of formulating a cover story, we settled on the manipulations we presented in the paper, but there could be more ways to elicit physical firmness. Future research on this topic could look at different sources of physical firmness and compare their effectiveness in eliciting structural alignment.

Beyond addressing the limitations, there are three directions that future research on the topic of structural alignment and physical firmness could investigate. These are understanding why experiencing physical firmness would lead to structural alignment, when physical firmness does not elicit structural alignment, and whether squishiness or the feeling of release from muscle contraction could elicit the opposite of structural alignment.

With regards to why experiencing physical firmness would lead to structural alignment. We speculate on two ways to answer this question. First, this increased correlation could mean that people are less able to differentiate between similar beliefs due to reduced cognitive capacity. Secondly, it could mean that a more abstract mindset is activated when experiencing physical firmness. If the first instance is correct, there is some evidence in the literature that temporarily lowered cognitive capacity leads to worse physical performance on exertion tasks^31^ (see also^32^ for an unsuccessful replication). However, in our studies, people experiencing physical firmness did not particularly exert themselves or experience much tiredness. Moreover, there is no clear evidence that habitual physical activity can benefit or harm cognitive functions^33^, although it is rare that studies test cognitive capacity during or shortly after exercise. One possible speculation is that participants experiencing physical firmness entered a mindset of action readiness and that nuanced thinking, required to differentiate similar beliefs, is less likely to occur when people are ready to act. Another possible speculation is that physical firmness would lead participants to adopt a more abstract mindset^34^, which would lead them to focus on the similarities between related beliefs and not the differences. In both cases, the influence physical firmness has on beliefs is indirect and may be metacognitive.

There may be moderating circumstances that reduces the effect of physical firmness on structural alignment. In our experiments, participants experienced a moderate amount of physical firmness that was not registered as being painful or tiring. However, if participants were asked to intensely firm their muscles, this could have led to feelings of discomfort or tiredness, which could have distracted participants from the feeling of firmness. Thus, intensity could be an important moderator to investigate to shed further light on the robustness of the effects of firmness.

Finally, squishiness or relaxation after firmness could be of interest when exploring other variables that influence structural alignment. Specifically, holding a soft toy in our hands or experiencing the feeling of relaxation after isometrically contracting our muscles might lead to the opposite effect of structural alignment. It might occur that beliefs that are structurally related but different in nature might be considered even further apart when experiencing squishiness, such that the correlation between these beliefs might be lower than when otherwise in a muscle neutral state. Such question remains open for further investigation and should help identify the full range of influences that muscle contraction or relaxation may have on higher order cognition.

In sum, this research contributes to knowledge and studies suggesting a connection between physical sensations and higher order cognition. Beliefs can shape judgments and evaluations and can influence behavior. Therefore, it is crucial to understand how and why simple bodily sensations, such as muscle contractions could affect how beliefs interact. While this research investigates the sensation of muscle firmness, another possibility is that the experience of malleability (e.g., by holding a squishy or soft object) could lessen structural alignment and reduce certainty in a particular belief structure. From the daily experience of contracting our jaw to possibly waving our fists in frustration against an oppressive system during a protest, these bodily sensations can affect our beliefs in ways that we just started uncovering.

## Data Availability

The datasets generated and the syntax files used to analyze the current studies are available in the Open Science Framework repository, https://osf.io/8e3cu/?view_only=ef0ad82c62e64f77afeb5bdd192bdeed.
